# β_2_-adrenergic agonists modulate TNF-α induced astrocytic inflammatory gene expression and brain inflammatory cell populations

**DOI:** 10.1186/1742-2094-11-21

**Published:** 2014-01-30

**Authors:** Guy Laureys, Sarah Gerlo, Anneleen Spooren, Frauke Demol, Jacques De Keyser, Joeri L Aerts

**Affiliations:** 1 Department of Neurology, University Hospital Brussels, Center for Neurosciences, Vrije Universiteit Brussel, Laarbeeklaan 101, 1090 Brussels, Belgium; 2 VIB Department of Medical Protein Research, Ghent University Department of Biochemistry (Faculty of Medicine and Health Sciences), Albert Baertsoenkaai 3, 9000 Ghent, Belgium; 3 Department of Physiology, Laboratory of Eukaryotic Gene Expression and Signal Transduction, Ghent University, Proeftuinstraat 86, 9000 Ghent, Belgium; 4 Department of Neurology, University Medical Center Groningen, RUG, Hanzeplein 1, 9713 GZ Groningen, the Netherlands; 5 Laboratory of Molecular and Cellular Therapy, Vrije Universiteit Brussel, Laarbeeklaan 103, 1090 Brussels, Belgium

**Keywords:** Astrocytes, β_2_-adrenergic receptors, Neuroinflammation, NF-κB

## Abstract

**Background:**

The NF-κB signaling pathway orchestrates many of the intricate aspects of neuroinflammation. Astrocytic β_2_-adrenergic receptors have emerged as potential regulators in central nervous system inflammation and are potential targets for pharmacological modulation. The aim of this study was to elucidate the crosstalk between astrocytic β_2_-adrenergic receptors and the TNF-α induced inflammatory gene program.

**Methods:**

Proinflammatory conditions were generated by the administration of TNF-α. Genes that are susceptible to astrocytic crosstalk between β_2_-adrenergic receptors (stimulated by clenbuterol) and TNF-α were identified by qPCR-macroarray-based gene expression analysis in a human 1321 N1 astrocytoma cell line. Transcriptional patterns of the identified genes *in vitro* were validated by RT-PCR on the 1321 N1 cell line as well as on primary rat astrocytes. *In vivo* expression patterns were examined by intracerebroventricular administration of clenbuterol and/or TNF-α in rats. To examine the impact on the inflammatory cell content of the brain we performed extensive FACS analysis of rat brain immune cells after intracerebroventricular clenbuterol and/or TNF-α administration.

**Results:**

Parallel transcriptional patterns *in vivo* and *in vitro* confirmed the relevance of astrocytic β_2_-adrenergic receptors as modulators of brain inflammatory responses. Importantly, we observed pronounced effects of β_2_-adrenergic receptor agonists and TNF-α on IL-6, CXCL2, CXCL3, VCAM1, and ICAM1 expression, suggesting a role in inflammatory brain cell homeostasis. Extensive FACS-analysis of inflammatory cell content in the brain demonstrated that clenbuterol/TNF-α co-administration skewed the T cell population towards a double negative phenotype and induced a shift in the myeloid brain cell population towards a neutrophilic predominance.

**Conclusions:**

Our results show that astrocytic β_2_-adrenergic receptors are potent regulators of astrocytic TNF-α-activated genes *in vitro* and *in vivo*, and ultimately modulate the molecular network involved in the homeostasis of inflammatory cells in the central nervous system. Astrocytic β_2_-adrenergic receptors and their downstream signaling pathway may serve as potential targets to modulate neuroinflammatory responses.

## Background

Neuroinflammation constitutes an immune response against a diverse spectrum of noxious insults in the central nervous system (CNS), including pathogen invasion, tissue damage, or neurodegenerative processes. In most situations, it provides the CNS with defense and repair mechanisms critical for its survival. However, immune cells can also add insult to injury in cases of chronic neuroinflammation or uncontrolled “auto”-immunity [1].

Astrocytes emerge as key players in the pathology of several neurological diseases [2] and hold promise as targets for modulating neuroinflammation in devastating diseases such as multiple sclerosis (MS) [3], amyotrophic lateral sclerosis [4], Parkinson’s disease [5], and Alzheimer’s disease [6]. Moreover, astrocytes play a major role in brain homeostasis by regulating energetic metabolism and neurotransmission, secretion of neurotrophic molecules, and expression of inflammatory molecules. Importantly, all these astrocyte functions seem to be regulated by astrocytic β_2_-adrenergic receptors [7].

The transcription factor NF-κB is a crucial regulator of immunity and inflammation and has been shown to be pivotal for astrocytic neuroinflammatory responses [8]. Targeted astrocytic NF-κB silencing reduced inflammatory processes associated with spinal cord injury and increased recovery [9]. Furthermore, in experimental allergic encephalomyelitis (EAE) neuroinflammation was reduced when NF-κB expression was downregulated in astrocytes [10].

The anti-inflammatory effects of β_2_-adrenergic receptors have been extensively studied in the pulmonary system and were mainly attributed to inhibition of NF-κB-dependent gene expression [11,12]. Furthermore, in astrocytes, β_2_-adrenergic receptors appear to play a prominent role in regulating NF-κB activity. In cultured astrocytes, activation of β_2_-adrenergic receptors, through enhancing cAMP, increased IκBα gene expression, which in turn reduced NF-κB activation either by maintaining NF-κB in the cytoplasm or by binding to NF-κB within the nucleus [13].

Tumor necrosis factor-α (TNF-α) has been documented as a cytokine that plays a critical role in neuroinflammation linked to neurodegeneration in a number of diseases, including MS, Parkinson’s, and Alzheimer’s disease; many of its effects are mediated by NF-κB activation [14]. The crosstalk between β_2_-adrenergic receptors and the NF-κB-dependent pathways in astrocytes upon proinflammatory TNF-α treatment seems more complex since we previously observed, *in vitro*, that co-treatment of β-adrenergic agonists and TNF-α resulted in a reduced expression of certain NF-κB‒dependent genes (e.g., ICAM-1), but also reinforced expression of other NF-κB‒dependent genes (e.g., IL-6) [15].

The aim of this study was to further dissect the *in vitro* and *in vivo* crosstalk between astrocytic β_2_-adrenergic receptors and NF-κB-dependent genes.

## Methods

### Cell culture

The human astrocytoma cell line 1321 N1 was a kind gift from Prof. Dr. Müller (University of Bonn). 1321 N1 cells were maintained in Dulbecco’s modified Eagle’s medium (DMEM), supplemented with 10% Fetal Calf Serum (FCS), 100 U/mL penicillin, and 100 μg/mL streptomycin (all from Invitrogen, Carlsbad, CA, USA). Cells were maintained at 37°C in a humidified atmosphere of 5% CO_2_. Cells were passaged using 0.05% (w/v) trypsin in 0.4% (w/v) EDTA.

Primary cultures of rat astrocytes were prepared from postnatal day 1 Wistar rats. All animal procedures were conducted in strict accordance with national guidelines and regulations on animal experiments and approved by the Ethics Committee on Animal Experiments of the Faculty of Medicine and Pharmacy of the Vrije Universiteit Brussels, Belgium. Briefly, after brain dissection, the brain hemispheres were mechanically dissociated under sterile conditions in phosphate buffered saline (PBS). After a centrifugation and washing step at 1,000 rpm for 5 min, cells were resuspended in culture medium (DMEM + glutaMAX + 10% fetal bovine serum (FBS) + 1% Pen Strep + 1% Fungizone) and residual tissue aggregates were removed by filtration through a 70-μm pore size cell strainer. The cells were plated in cell culture flasks (about 1.5 brains/flask) and grown in a humidified atmosphere of 5% CO_2_ air at 37°C. The medium was changed weekly until a confluence of 80% was attained. Cell culture flasks were then incubated in a shaker at 180 rpm to remove any residual oligodendrocytes and microglia. After 6 h, the medium was changed (discarding the non-adherent cells). Cells were grown for another 18 h for a total of 24 h incubation and medium was changed until cells had grown to confluence. Previous studies have determined the high degree of astrocyte purity (~95%) with this culture technique [16]. Two or three days later, cells were collected after trypsinization and distributed at a concentration of 100,000 cells in a 6-well plate in 2 mL of culture medium (+ 10% FCS) for RT-PCR. After adherence (48 h), cell maturation was initiated by decreasing the FBS concentration to 3% for 7 days.

### *In vitro* treatment protocol

*In vitro* procedures for the human astrocytoma cell line and primary rat astrocytes were similar except for the dosage of TNF-α (2000 IU/mL of human TNF-α produced at the Dept. of Biomedical Research of UGent) and 10 ng/mL rat TNF-α (Sigma-Aldrich, St. Louis, MO, USA), clenbuterol (Sigma-Aldrich) was administered at 10 μM for human and rat astrocytes. After 2 h of starvation on DMEM/1% FCS/Pen-Strep, cells were exposed to the different stimuli for 3 h: vehicle, clenbuterol, TNF-α, and TNF-α + clenbuterol. Cells were washed with ice-cold PBS and resuspended in 350 μL Qiagen RNeasy lysis buffer (RLT + β-ME) before homogenization with the Qiashredder. Ethanol (350 μL) was added and lysates were stored at -80°C until further processing.

### Animals and surgery

Male albino Wistar rats (Charles River Laboratories, Brussels, Belgium), weighing 260–320 g, were housed in groups of 4 for at least 7 days following arrival in the animal facilities. Standard environmental conditions were ensured (temperature 21°C, humidity 60%, 10/14 h dark/light cycle, lights on at 07:00 am). For surgery, rats were anaesthetized with a mixture of ketamine hydrochloride and diazepam (90.5:4.5 mg/kg). A CMA/12 guide cannula with a replaceable inner guide (CMA Microdialysis, Solna, Sweden) was implanted stereotactically in the left lateral ventricle. The coordinates were 1.4 mm lateral and 0.9 mm posterior to bregma and 3.5 mm ventral starting from the dura mater [17]. Correct placement of the cannula was confirmed by visual identification of cerebrospinal fluid after the insertion of the cannula. The cannula was fixed to the skull with dental acrylic cement. After surgery, the animals were housed in experimental cages with access to water and standard laboratory chow *ad libitum* to recover overnight from surgery.

### *In vivo* treatment protocol

The guide cannula obturator was replaced with a CMA/12 microdialysis probe (CMA Microdialysis) trimmed down for intracerebroventricular (ICV) injection just before the start of the experiment. Aqueous solutions were made with purified water (Seralpur pro 90 CN, Belgolabo, Overijse, Belgium) and filtered through a 0.2-μm membrane filter. The aqueous medium for ICV administration, further defined as Ringer’s solution, consisted of 147 mM NaCl, 1.1 mM CaCl_2_, and 4 mM KCl. TNF-α and/or clenbuterol dissolved in Ringer’s solution were administrated at a rate of 1 μL/min by means of a CMA-100 pump for a total dose of 200 ng (10 μg/100 μL) of TNF-α and clenbuterol for a total dose of 20 μg (10 μg/μL). Rats were euthanized 3 hours after ICV-administration, after which the whole brain was quickly removed and ‘snap-frozen’ in liquid nitrogen before storage at -80°. The four experimental groups consisted of a non-operated control group (n = 6), a sham group with ICV Ringer’s administration (n = 6), a clenbuterol 20 μg ICV group (n = 6), a TNF-α 200 ng ICV group (n = 6), and a group with clenbuterol 20 μg and TNF-α ICV co-administration (n = 6). The same protocol was used for the FACS experiments; however, rats were kept overnight with removal of the brain after 24 h. This time point was chosen based on previous data showing inflammatory cell migration after 24 h in sheep upon ICV TNF-α administration [18]. Preliminary FACS experiments in rats confirmed a TNF-α response at 24 h. The treatment groups consisted of a sham group with ICV Ringer’s administration (n = 6), a clenbuterol 20 μg ICV group (n = 6), a TNF-α 200 ng ICV group (n = 5), and a group with clenbuterol 20 μg and TNF-α ICV co-administration (n = 5).

### qPCR array

We employed a qPCR-based gene expression system that measures the expression of 96 NF-κB dependent immunological genes (StellARray, Lonza, Basel, Switzerland). These genes are recognized for having both κB binding sites in the promoter and gene expression changes associated with increased NF-κB activity. The genes included in the array encode diverse proteins such as cytokines, chemokines, complement proteins, immunological receptors, and transcription factors (for the complete list, see Additional file 1: Table S1). We performed the qPCR array on three biological replicates for each of the following inductions (6 h treatments): untreated (vehicle), isoproterenol only (Sigma-Aldrich), TNF-α only, and isoproterenol + TNF-α. This initial exploration was performed with isoproterenol, known to be a mixed β_1_ and β_2_ agonist. However, the 1321 N1 cell-line expresses only the β_2_-subtype [19]. For confirmation with RT-qPCR and rat studies the selective β_2_ agonist clenbuterol was preferred excluding effects mediated by other β-adrenergic subtypes.

### RNA isolation and quantitative real-time PCR

Total RNA from rat brain was isolated using Trizol reagent (Invitrogen). Briefly, complete rat hemispheres were homogenized in Trizol reagent using a tissue homogenizer. Total RNA was isolated from these homogenates according to the manufacturer’s instructions. To remove any gDNA contaminating the RNA samples, a DNase treatment was performed. Total RNA from 1321 N1 cells and rat astrocytes was prepared using the RNeasy Mini kit (Qiagen) including an on-column DNase digestion step according to the manufacturer’s instructions. Reverse transcription was performed on 0.5 μg of total mRNA using the Verso cDNA kit (Thermo Fisher, Surrey, UK). For real-time cDNA amplification we used SYBR Green Mastermix (Bio-Rad or Roche) and the primers as stated in Additional file 1: Table S2 of the supplementary materials. The qPCR array and *in vivo* samples were analyzed using the BioRad iCycler (Bio-Rad, Hercules, CA, USA). All *in vitro* samples were analyzed on the LightCycler (Roche Applied Science, Penzberg, Germany). A serial dilution of a cDNA mix standard, representing a pool of cDNAs obtained from representative samples, was used to determine the efficiency of the PCR reaction and to calculate relative mRNA inputs. Cycle threshold (C_T_) values for each gene were normalized to those for hypoxanthine guanine phosphoribosyl transferase (HPRT).

### Flow cytometric analysis of brain tissue

Isolation of brain infiltrating leukocytes was performed as described by Beeton and Chandy [20]. Under deep pentobarbital-anesthesia, rats underwent cardiac perfusion with saline for 15 min to minimize contamination from intravascular white blood cells. After decapitation, the brain was quickly removed and placed in a 50 mL tube containing ice-cold PBS. Subsequent to mechanical dissociation, brain cells were passed through a 70-μm cell strainer and the cell suspension was collected into a 50 mL tube on ice. After centrifugation for 8–10 min at 390 *g*, cells were resuspended in 20 mL PBS + 30% Percoll and overlaid onto 10 mL of PBS + 70% Percoll. This gradient was centrifuged at 390 *g* for 20 min at room temperature. After removal of the fat on top of the suspension, cells were collected from the interface and washed twice with PBS. The cell content was then counted and dilutions were performed in order to obtain 1–2 × 10^6^ cells per tube for FACS analysis. After centrifugation at 1,400 rpm for 3 min, the supernatant was discarded and the pellet incubated with antibody mixture for 30 min at 4°C. Next, cells were washed in FACS buffer (PBS/BSA/Azide) (400 μL) and then resuspended in 400 μL of clean FACS buffer. Dilutions for FACS antibodies were as follows: CD45 AlexaFluor 700 (BioLegend, 1:50), CD3 FITC (BioLegend, 1:100), CD4 PECy7 (BioLegend, 1:400), CD8a PERCP (BioLegend, 1:50), CD45R PE (eBioscience, 1:50), CD161 APC (BioLegend, 1:400), CD163 FITC (Acris, 1:20), CD11b/c APC (BioLegend, 1:50), and His45 PE (eBioscience, 1:200). For each condition, three separate samples were prepared for T cell staining (CD45/CD3/CD4/CD8), for lymphocyte staining (CD45/CD3/CD4/CD8/CD45R/CD161), and for myeloid cell staining (CD45/CD163/CD11b/c/His45), respectively. Samples were collected on an LSR Fortessa flow cytometer (BD Biosciences) and analysis of results was performed using FACSDiva Software (BD Biosciences).

### Statistical analysis

The qPCR-array data were analyzed using Global Pattern Recognition™ software (GPR) (Lonza, Basel, Switzerland). This software does not use standard reference genes for normalization, but instead makes an analysis of the global expression pattern searching for consistency in the data. The expression level of each gene is globally positioned with respect to the expression levels of all the other genes within an experiment and based on this an algorithm subsequently normalizes the gene expression data. A control for genomic DNA contamination is furthermore included and a conventional ∆∆Ct analysis using 18S as a normalizer gene is additionally displayed. For more background and validation we refer to the 2003 paper by Akilesh et al. [21]. We subsequently analyzed which genes in the isoproterenol + TNF-α condition were changed in a way that could not be predicted by combining the information from the vehicle versus isoproterenol and vehicle versus TNF-α comparisons. These genes make up the two subsets that show inhibitory or synergistic crosstalk as shown in Table 1. Data for RT-qPCR and FACS analysis are presented as median ± interquartile range. qPCR and FACS data were analyzed with a Kruskal-Wallis test followed by Dunn’s test for pairwise comparisons (*P <0.05, **P <0.01, and ***P <0.0001). Statistical analysis was performed with the InStat Prism statistical package (Prism 5 for Mac OS X, GraphPad Software, La Jolla, USA).

**Table 1:** **Genes showing crosstalk between the β**_**2**_**-adrenergic and TNF-α triggered pathways**

	**Fold induction versus vehicle**			***P *** **value**
GENE	**ISO**	**TNF**	**TNF + iso**	**ISO + TNF vs. TNF**
**A20**	1.11	**25.70**	**70.24**	**0.03**
ABCB1	-1.09	**18.22**	**17.10**	0.694
**C3**	-1.10	**22.69**	**8.85**	0.113
**CCL5**	1.84	**89.53**	**49.39**	0.602
CSF2	-1.92	**55.06**	**17.88**	0.069
**CXCL2**	**5.57**	**10.34**	**29.12**	0.053
**CXCL3**	**3.17**	**13.08**	**35.17**	0.097
**ICAM-1**	-1.03	**39.16**	**16.01**	0.146
**IL-6**	2.18	1.66	**253.07**	**0.002**
LEF1	-4.75	**-52.41**	1.09	**0.015**
PSMB9	1.03	**12.72**	**8.73**	0.244
PTX3	-1.37	**21.11**	**14.45**	0.220
**VCAM-1**	1.07	**445.48**	**153.39**	0.510
BCL3	1.19	**2.70**	**2.92**	0.265
CSF1	-1.40	**4.38**	**4.48**	0.262
FAS	1.18	**3.80**	**5.99**	0.173
IL15	1.74	**6.20**	**5.61**	0.289
IL1RN	1.79	2.44	**3.01**	0.237
PLAU	**-2.69**	**12.97**	**16.09**	0.108
TAP1	1.25	**18.71**	**19.28**	0.245
TRAF1	2.19	**6.37**	**4.79**	0.424

Marked in bold are: fold inductions significant from vehicle-treated, significant *P* values for the GPR comparison of TNF + ISO vs. TNF and genes selected for further analysis.

## Results

### *In vitro* qPCR-array

We previously reported dual effects of β-agonist co-treatment on TNF-α-induced expression of selected prototypical NF-κB target genes [15]. To further explore this intriguing modulation of the NF-κB-dependent gene network by β-agonists in astrocytes, we here performed a qPCR-array-based gene expression analysis in human 1321 N1 astrocytoma cells.

The full dataset of the qPCR macro-array, investigating the expression of 96 validated NF-κB-dependent target genes, can be found in the Additional file 1: Table S1. An extract of these results, showing only the 21 genes for which at least one of the treatments (iso, TNF-α, or iso + TNF-α) resulted in significantly changed mRNA levels (as compared to vehicle) is represented in Table 1. As evident from this analysis, from the 96 NF-κB target genes that were included in the qPCR array, 18 genes were significantly upregulated and 1 gene (lymphoid enhancer-binding factor 1, *LEF1*) was significantly downregulated upon TNF-α treatment. Isoproterenol treatment significantly promoted the transcription of only two genes, chemokine (C-X-C Motif) Ligands 2 and 3 (*CXCL2* and *CXCL3*), and downregulated one (Urokinase-type plasminogen activator (*PLAU*)). Upon isoproterenol/TNF-α co-treatment, expression of two additional genes (*IL6* and IL-1 receptor agonist, *IL1RN*), for which the expression was not significantly changed by either TNF-α or β-adrenergic receptor agonist treatment alone, was upregulated. In addition, TNF-α-induced *LEF1* downregulation was no longer significant upon co-treatment with isoproterenol. Moreover, it is clear from this analysis that there are subsets of NF-κB target genes that respond differently to β-adrenergic receptor agonist co-treatment. For instance, although these changes did not reach statistical significance (cf. *P* values for TNF-α/iso co-treatment vs. TNF-α in Table 1), the TNF-α-induced expression of granulocyte-macrophage colony stimulating factor (*CSF2*), intracellular adhesion molecule-1 (*ICAM1*), C3 convertase (*C3*), proteasome subunit-beta type-9 (*PSMB9*), pentraxin-3 (*PTX3*), and chemokine (C-C motif) ligand 5 (*CCL5*) was inhibited, whereas that of TNF-α induced protein-3 (*TNFAIP3*), *LEF1*, *CXCL2*, interferon regulatory factor 1 (*IRF1*), *CXCL3*, *FAS*, and *PLAU* was enhanced upon β-adrenergic receptor agonist co-treatment. The expression of *RELB*, *IL15*, transporter associated with antigen processing 1 (*TAP1*), *CSF1*, and B-cell lymphoma 3-encoded protein (*BCL3*) did not change upon β-adrenergic receptor agonist co-administration as compared to the TNF-α only set-up.

### *In vitro* and *ex vivo* qPCR data

Based on the qPCR array data, we selected a number of gene candidates for validation in 1321 N1 cells and further *in vitro* and *in vivo* study in rats. We focused on eight genes for which there was a clear indication for crosstalk upon TNF/isoproterenol co-treatment (Table 1), and for which reliable primer sets for SYBR green qPCR amplification of the rat and human cDNAs could be designed (A20 (TNFAIP3-gene), C3, CCL5, CXCL2, CXCL3, ICAM1, IL-6, and VCAM1).

**Figure 1:**
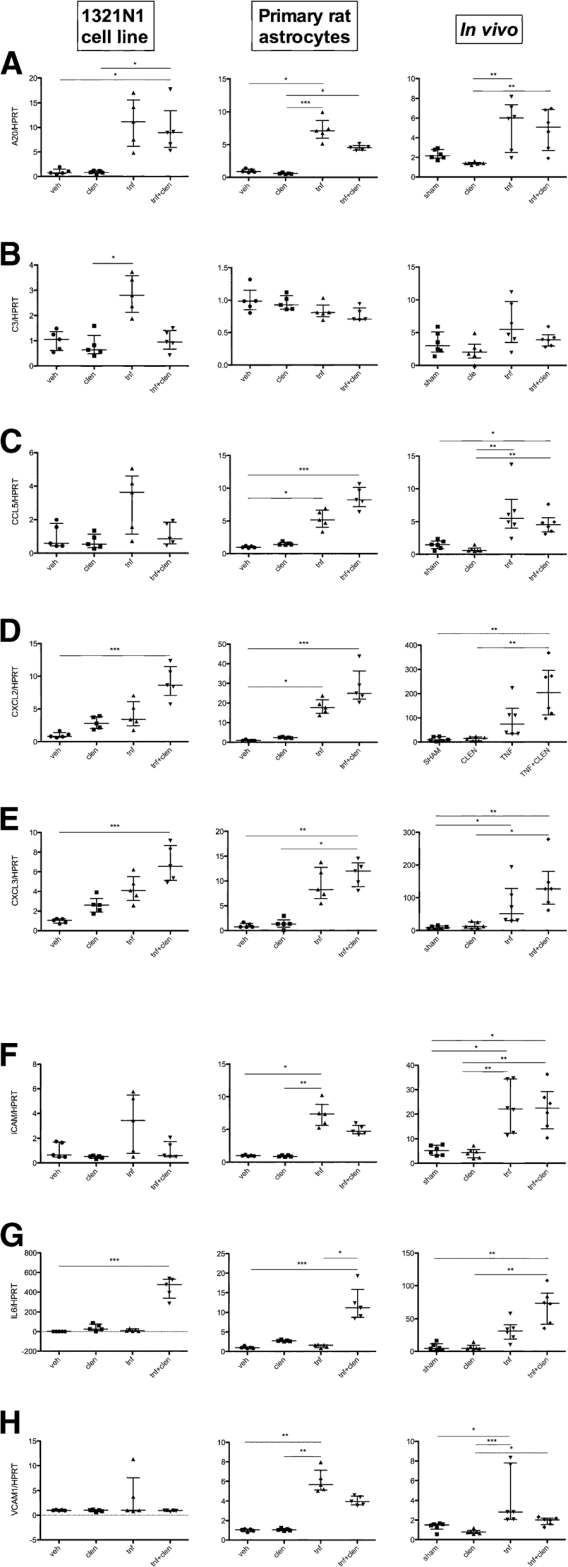
***In vitro *and *in vivo *data from RT-qPCR for the 1321 N1 cell line validation, *in vitro *rat astrocyte and *in vivo *experiments.** (Kruskal-Wallis with Dunn’s post-hoc analysis, *P <0.05, **P <0.01, and ***P <0.001). Graphs represent fold expression after vehicle, clenbuterol, TNF-α and clenbuterol with TNF-α co-administration. All data are plotted as median with interquartile range for the following genes: **(A)** A20, **(B)** C3, **(C)** CCL5, **(D)** CXCL2, **(E)** CXCL3, **(F)** ICAM1, **(G)** IL-6 and **(H)** VCAM1.

We found that TNF-α induced A20 mRNA expression *in vitro* and *in vivo*, with a non-significant trend towards suppression by clenbuterol co-administration that was most pronounced in the primary rat astrocyte cells (Figure 1A). Although not significant, a tendency for antagonism of TNF-α-mediated C3 expression that was observed upon co-treatment of 1321 N1 cells, was also apparent *in vivo*. The transcription of C3 by primary rat astrocytes was, however, not significantly affected by any of the treatments (Figure 1B). Data on the expression of the chemokine CCL5 (RANTES) were somewhat contradictory (Figure 1C). The human astrocytic cell-line showed a non-significant trend towards an antagonism, whereas a significant potentiation was detected in the primary rat astrocytes. On the other hand, *in vivo* clenbuterol treatment showed a non-significant trend towards suppression of sham-intervention- and TNF-α-mediated expression of CCL5. For the chemokines CXCL2 and CXCL3, a synergistic upregulation was observed, both *in vitro* and *in vivo*, with clenbuterol and TNF-α co-administration (Figure 1D, E). Figure 1G demonstrates β_2_-adrenergic potentiation for the TNF-α-induced expression of IL-6 in human and rat astrocytes both *in vitro* and *in vivo*. For the adhesion molecules ICAM1 and VCAM1 (Figure 1F,H), a TNF-α-mediated induction was observed both *in vivo* and *in vitro*, and this effect was counteracted by clenbuterol co-administration, an effect that was most pronounced for VCAM1 (significances as stated in the figures).

### *Ex vivo* FACS analysis of inflammatory cell populations

**Figure 2:**
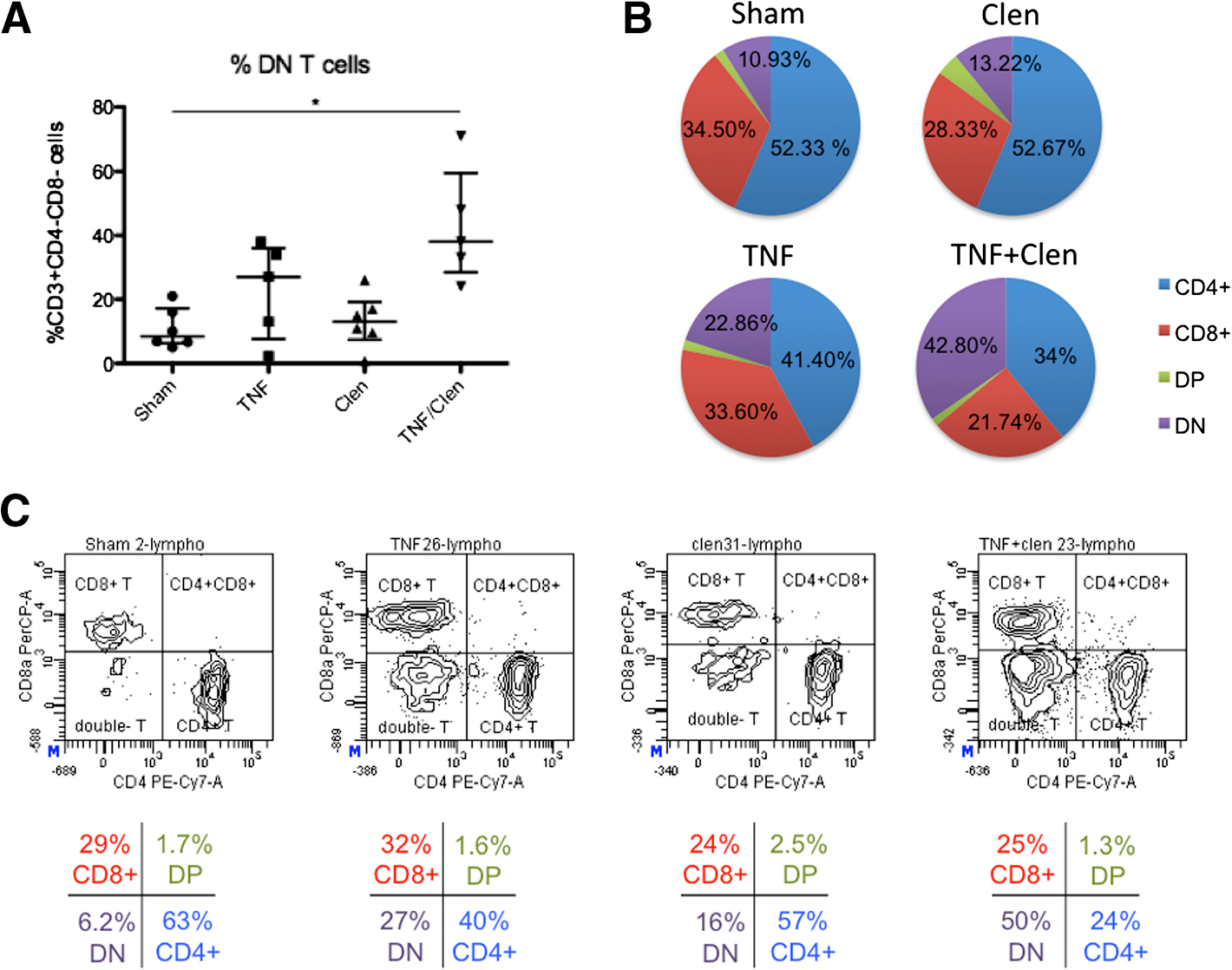
**FACS analysis of the rat brain T cell population after TNF-α and/or clenbuterol administration. (A)** TNF-α/clenbuterol co-administration induces a significant increase in the proportion of CD4^-^CD8^-^ double negative T cells (Kruskal-Wallis with Dunn’s post-hoc analysis, * *P* <0.05). **(B)** Pie charts of the composition of the T cell repertoire under the different treatment conditions. **(C)** Representative FACS-plots for the different treatment conditions. Color code: red for CD3^+^CD4^-^CD8^+^ T cells, blue for CD3^+^CD4^+^CD8^-^ T cells, green for CD3^+^CD4^+^CD8^+^ double positive T cells, purple for CD3^+^CD4^-^CD8^-^ double negative (DN) T cells.

**Figure 3:**
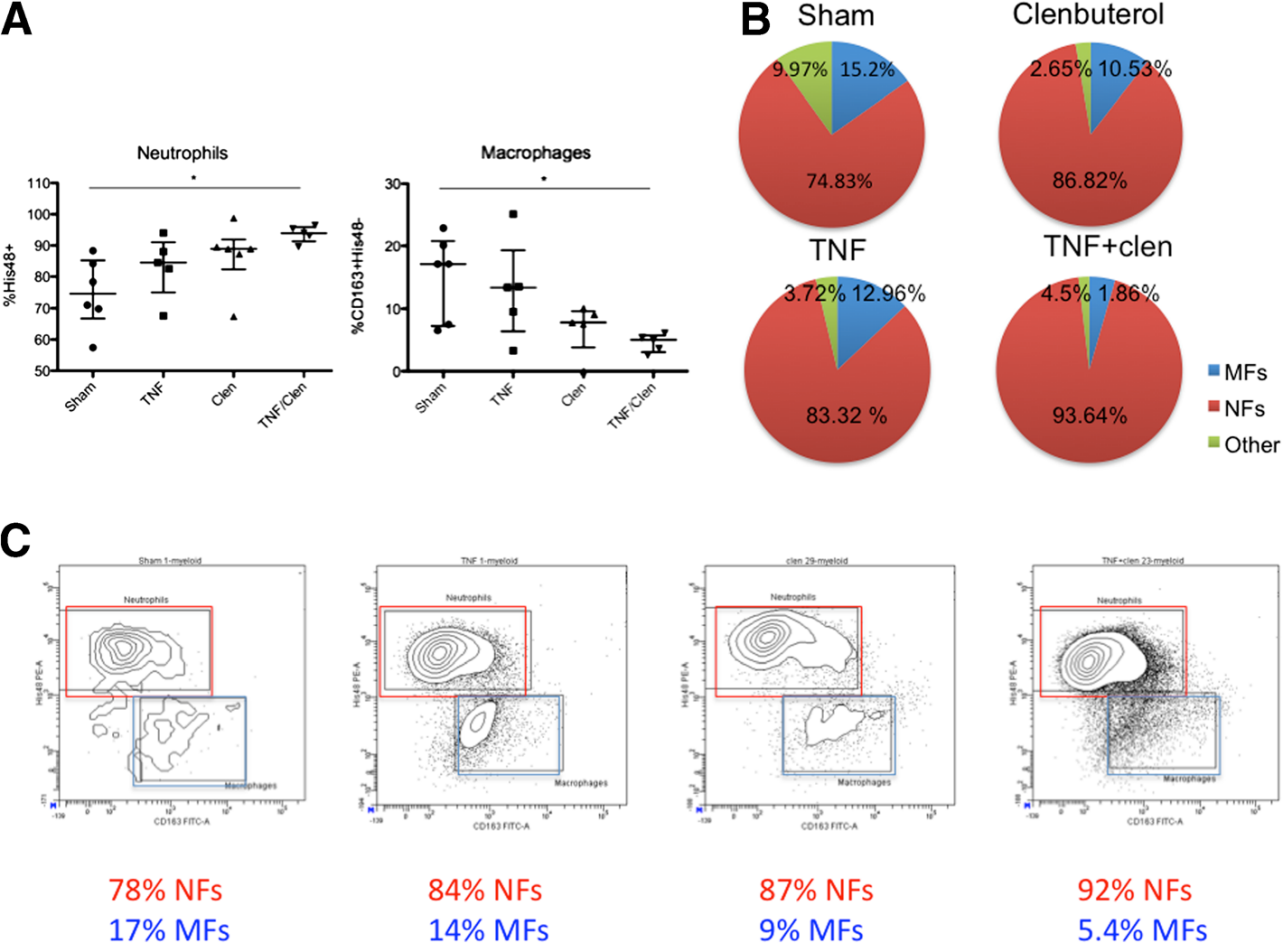
**FACS analysis of the rat brain myeloid cell population after TNF-α and/or clenbuterol administration. (A)** TNF-α/clenbuterol co-administration induces a significant increase in the proportion of neutrophils and a significant decrease in macrophages (Kruskal-Wallis with Dunn’s post-hoc analysis, *P <0.05). **(B)** Pie charts of the composition of the myeloid repertoire under the different treatment conditions. **(C)** Representative FACS-plots for the different treatment conditions. Color code: red for macrophages (MFs), blue for neutrophils [22], green for other myeloid cells.

Several of the genes for which we found that the expression was affected by TNF-α/clenbuterol co-treatment play a role in leukocyte chemotaxis. We therefore evaluated, via FACS analysis, whether the distribution of leukocyte subsets was changed in the brain during the different treatment paradigms. Representative plots showing the gating strategy for lymphocyte and myeloid lineages are depicted in Additional file 1: Figures S1 and S2, respectively. A summary of all analyzed subsets can be found in Additional file 1: Figure S3. The total percentage of leukocytes (CD45^+^ cells) was not significantly changed over the different treatment conditions. Proportions of CD3^-^CD161^high^CD45R^-^ (NK cells) and CD3^+^CD161^+^ (NKT cell) populations were also not significantly altered after TNF-α and/or clenbuterol stimulation. A non-significant trend (significant Kruskal-Wallis at *P* = 0.0294, non-significant Dunn’s multiple comparison test) towards reduced numbers of B cells (CD3^-^CD161^-^CD45R^+^) after clenbuterol and TNF-α/clenbuterol co-treatment can be noted. Within the T cell population (Figure 2), TNF-α/clenbuterol co-administration led to a significant increase in the CD4^-^CD8^-^ double negative phenotype. The myeloid cells showed a significant decrease in the proportion of macrophages and an increase in neutrophils under the influence of clenbuterol and TNF-α administration, with a significant shift towards a neutrophilic predominance with TNF-α/clenbuterol co-treatment (Figure 3).

## Discussion

Previous publications demonstrated that activation of β_2_-adrenergic receptors enhances TNF-α-induced expression of IL-6 in both rat astrocytes and the human 1321 N1 astrocytoma cell line [15,23]. This was confirmed in our *in vitro* qPCR data. Confirmation of these effects *in vivo* has been lacking. We show, for the first time, by ICV administration of both TNF-α and clenbuterol in rats, that this also occurs *in vivo*. IL-6 plays an ambiguous role in the CNS, with neurotrophic and neuroprotective effects on the one hand, and destructive effects inducing demyelination and astrogliosis on the other [24]. Our results apparently contradict a previous study suggesting a suppressive effect of β_2_-adrenergic receptor activation on IL-6 expression *in vivo*, as witnessed by decreased IL-6 expression in astrocytes after locus coeruleus lesioning [25], as well as a more recent study showing that clenbuterol suppresses IL-6 expression in rat cortex after systemic lipopolysaccharide (LPS) administration [26]. In the first study there was no pro-inflammatory environment. In the latter study, in which LPS as well as clenbuterol were administered systemically, results might have been confounded by indirect systemic effects which were excluded by our ICV approach. Indeed, systemic administration of LPS increased plasma IL-6, an effect counteracted by intraperitoneal clenbuterol administration [27]. In addition, although both LPS and TNF-α use NF-κB as an essential signaling mediator, they also induce non-redundant signaling cascades that might explain differences in the outcome of crosstalk with other signaling cascades.

In accordance with the previously described anti-inflammatory action of β-adrenergic receptor agonists, we found that TNF-α-induced expression of ICAM1 and VCAM1 adhesion molecules was antagonized by clenbuterol co-treatment *in vitro*. However, this inhibitory effect could not be demonstrated *in vivo.* One possibility is that local inhibitory responses occur *in vivo*, but that these are masked by the expression of ICAM1 and VCAM1 by cells that do not respond to the clenbuterol treatment in the same manner as astrocytes. A recent study documented a suppressive action of noradrenaline reuptake inhibitors on CAM expression *in vivo*, which was due to increased noradrenaline availability at glial cells [28], suggesting a potential role in regulating inflammatory cell migration across the blood brain barrier. Our data point to the astrocytic β_2_-adrenergic receptors as possible effectors of noradrenaline action in regulating inflammatory cell migration across the blood brain barrier.

One of the most remarkable findings of this study was the susceptibility of different chemokines to TNF-α/β_2_-adrenergic receptor interaction *in vitro* and *in vivo*. These chemokines have a specific tropism for attracting immune cells. CXCL2 mainly attracts polymorphonuclear leukocytes, CXCL3 controls migration and adhesion of monocytes, and CCL5 is chemotactic for T cells, eosinophils, and basophils [29].

Although it has been previously reported that β-adrenergic receptor activation inhibits NF-κB activity by enhancing the expression of the NF-κB inhibitor IκB in astrocytes [13], it is difficult to reconcile such a global NF-κB inhibitory mechanism with our data, showing gene-selective effects of β-agonists. In line with this, we reported that, in 1321 N1 astrocytes, the expression of selected NF-κB target genes was inhibited without apparent changes in IκB levels, indicating additional regulatory mechanisms must exist [15]. Multiple studies have indicated that activation of the 3′-5′-cyclic adenosine monophosphate (cAMP) – protein kinase A (PKA) – cAMP response element binding protein (CREB) pathway, which is the canonical signaling cascade induced by β-agonists, leads to the competition of active CREB with NF-κB for the limiting co-activator CREB-binding protein (CBP). As CREB has a higher affinity for CBP than NF-κB, the result of this is that the expression of NF-κB target genes, which use CBP as a cofactor, is inhibited upon activation of CREB [30]. Selected NF-κB target genes, such as IL-6 [15] and CXC chemokines [31], have binding sites for both CREB and NF-κB in their promoters and these genes appear to be targets for potentiation, rather than inhibition, by β-agonists. The exact molecular details of the selective regulation of NF-κB target genes by β-agonists will, however, require further study.

It has been suggested that TNF-α plays an important role in attracting leukocytes towards the brain in diseases as diverse as stroke, HIV-encephalitis, and MS [32-36]. Astrocytic NF-κB has been shown to play a major role in chemokine-dependent attraction of leukocytes as a result of traumatic brain injury [37]. In EAE experiments, it has been demonstrated that astrocytic NF-κB modulates chemokine, cytokine and adhesion-molecule expression, CNS inflammatory cell migration, and ultimately clinical outcome [10]. Since all of the studied NF-κB dependent molecules in our experiments have pleiotropic effects on the myriad of resident brain and immune cells, it is impossible to predict the exact outcome of interventions on neuroinflammatory cell populations. It is, however, remarkable that TNF-α/clenbuterol co-administration shifts the myeloid brain cell population towards a neutrophilic predominance. This correlates well with the *in vitro* and *in vivo* potentiation of TNF-α/clenbuterol co-treatment on astrocytic CXCL2 expression that we observed. CXCL2 is a powerful chemo-attractant drawing neutrophils towards the CNS [38-40]. Astrocytes have been identified as a source of CXCL2 involved in CNS neutrophil migration during early inflammatory responses in mouse spinal cord injury [41].

Another prominent finding in our FACS data is that TNF-α/clenbuterol co-treatment results in a shift towards CD4^-^CD8^-^ double negative (DN) T cells, expanding from 11% (sham) to 43% (TNF-α/clenbuterol) of the T cell population. These enigmatic cells have been identified as a marginal population in mice and humans, comprising about 1% to 3% of the total T cell pool [42,43]. DNT cell prevalence seems to be organ- and inflammation-dependent (for review see [44]). This subset is thought to act as a regulatory T cell population implicated in counteracting allograft rejection, graft-versus-host disease, and autoimmune processes [45,46]. The origins and activation mechanisms of this peculiar subset remain unclear, although it has been shown that extrathymic conversion from CD4^+^ T cells [47] can give rise to potent immunoregulatory DNT cells and CD8^+^ T cells that have the ability to convert to a DN phenotype [48].

The importance of the shift in neutrophilic predominance over macrophages is more difficult to interpret. Although crucial for clearance of infectious agents, their role in neuroinflammatory conditions remain unclear. In EAE, neutrophil depletion seems to have a protective effect [49], although neutrophils are not detected in MS lesions [50]. In neuromyelitis optica, an antibody-mediated inflammatory condition mainly affecting spinal cord and optic nerves, neutrophils are abundant in lesion pathology [51]. Recent data from stroke research [52] suggest that neutrophils may also have a neuroprotective phenotype (the so called “N2”-phenotype) and that this phenotype may be stimulated by the NF-κB inhibitory pathway PPAR-γ [53]. The potential of NF-κB inhibition in inducing neuroprotective neutrophils deserves further attention.

To the best of our knowledge, we are the first to describe the presence of the DNT subset in rat brain and its upregulation by TNF-α and β_2_-adrenergic receptor co-treatment. We previously reported that β_2_-adrenergic receptors are selectively downregulated in astrocytes in MS [54]. This downregulation might play a role in the neurodegenerative aspect of the disease [2], but it remains unclear how it can explain the inflammatory aspect of MS. A decrease in immunoregulatory DNT cells may be a component linking downregulation of astrocytic β_2_-adrenergic receptors with neuroinflammation in MS. It should be stressed that these findings are preliminary and that the mechanisms behind these shifts in T cell subsets and the potential role of β_2_-adrenergic receptors in regulating CNS autoimmunity deserve further investigation.

## Conclusions

Treatment of neuroinflammation in CNS injury and degeneration remains a therapeutic dilemma. Our *in vitro* and *in vivo* data indicate that modulating the astrocytic β_2_-adrenergic receptor tone alters NF-κB-dependent effects and the immune cell content of the CNS in proinflammatory conditions.

## Abbreviations

BCL3: B-cell lymphoma 3-encoded protein; C3: C3 convertase; cAMP: 3′-5′-cyclic adenosine monophosphate; CCL5: Chemokine (C-C motif) ligand 5; CNS: Central nervous system; CREB: cAMP response element binding protein; CSF: Granulocyte-macrophage colony stimulating factor; CT: cycle threshold; CXCL2/3: Chemokine (C-X-C Motif) Ligand 2 and 3; DMEM: Dulbecco’s modified eagle’s medium; EAE: Experimental allergic encephalomyelitis; FACS: Fluorescence activated cell sorter; FBS: Fetal bovine serum; FCS: Fetal calf serum; HPRT: Hypoxanthine guanine phosphoribosyl transferase; ICAM1: Intracellular adhesion molecule-1; ICV: Intracerebroventricular; IRF1: Interferon regulatory factor 1; LEF1: Lymphoid enhancer-binding factor 1; MS: Multiple sclerosis; NF-κB: Nuclear factor kappa-light-chain-enhancer of activated B cells; PBS: Phosphate buffered saline; PCR: Polymerase chain reaction; PKA: Protein kinase A; PLAU: Urokinase-type plasminogen activator; PSMB9: Proteasome subunit-beta type-9; PTX3: Pentraxin-3; TAP1: Transporter associated with antigen processing 1; TNF-α: Tumor necrosis factor alpha; TNFAIP3: TNF-α induced protein-3; VCAM1: Vascular cell adhesion molecule 1.

## Competing interests

The authors declare that there are no conflicts of interests.

## Authors’ contributions

GL and JDK conceived the project; SG designed the *in vitro* and *in vivo* PCR experiments and JLA the FACS methodology. GL performed most of the experiments with AS, SG, and AD performing additional experiments. GL performed the statistical analysis with critical assistance of JDK and JLA. GL, SG, JDK, and JLA wrote the paper. All authors read and made comment on the manuscript during its drafting.

## Authors’ information

Guy Laureys and Sarah Gerlo share first authorship. Jacques De Keyser and Joeri L. Aerts share senior authorship.

## Supplementary Material

Additional file 1 **Table S1.** Genes screened for in the qPCR-array for each treatment condition. Significant changes in upregulation (*P* <0.05) are marked in red, significant downregulation in green (*P* <0.05), and unchanged genes in yellow versus vehicle. For clarity, genes with a significant change have been put alphabetically at the beginning of the table followed by unaltered genes in alphabetical order. **Table S2.** Primer sequences used for RT-qPCR. **Figure S1.** Representative plots illustrating lymphocyte gating. **Figure S2.** Representative plots illustrating myeloid gating. **Figure S3.** Leucocyte subsets plotted as median with interquartile range for the different treatment conditions. Statistical analysis was performed with a Kruskal-Wallis test with Dunn’s post-hoc for multiple comparisons (* = P < 0.05). Abbreviations: Natural Killer (NK) cells, Natural Killer T (NKT) cells, CD4 + CD8+ double positive (DP) T cells, CD4-CD8- double negative (DN) T cells. Click here for file
