# Supplementary material for: β2-adrenergic agonists modulate TNF-α induced astrocytic inflammatory gene expression and brain inflammatory cell populations
Source: J Neuroinflammation. 2014 Jan 30;11:21. doi: 10.1186/1742-2094-11-21 (PMC3942172; doi:10.1186/1742-2094-11-21)

Supplementary material:

Table1: Genes screened for in the qPCR-array. For each treatment condition significant changes upregulation (p<0,05) is marked in red, significant downregulation in green (p<0,05) and unchanged genes in yellow versus vehicle. For clarity genes with a significant change have been put alphabetically at the beginning of the table followed by unaltered genes in alphabetical order.

| Vehicle  vs  iso | | | Vehicle  vs  TNF-α | | Vehicle  Vs  Iso + TNF-α | |
| --- | --- | --- | --- | --- | --- | --- |
| **GENE** | **P-value** | **GPR FOLD CHANGE** | **P-value** | **GPR FOLD CHANGE** | **P-value** | **GPR FOLD CHANGE** |
| A20 | 0,43346 | 1,11279 | 0,00431 | 25,69676 | 0,00196 | 70,24346 |
| ABCB1 | 0,41182 | -1,09072 | 0,01868 | 18,22339 | 0,01926 | 17,1036 |
| BCL3 | 0,28225 | 1,18515 | 0,04858 | 2,69592 | 0,04574 | 2,92071 |
| C3 | 0,52742 | -1,10114 | 0,00532 | 22,69485 | 0,01454 | 8,84519 |
| CCL5 | 0,26918 | 1,83573 | 0,02702 | 89,52526 | 0,03659 | 49,39362 |
| CSF1 | 0,35526 | -1,39866 | 0,02722 | 4,37834 | 0,02731 | 4,47729 |
| CSF2 | 0,10146 | -1,91509 | 0,00157 | 55,06245 | 0,00438 | 17,87786 |
| CXCL2 | 0,00371 | 5,569086 | 0,01093 | 10,34074 | 0,00371 | 29,11855 |
| CXCL3 | 0,02314 | 3,17415 | 0,01274 | 13,08497 | 0,00375 | 35,17434 |
| FAS | 0,27512 | 1,17501 | 0,03752 | 3,80237 | 0,0168 | 5,98598 |
| ICAM-1 | 0,45269 | -1,03311 | 0,0028 | 39,15841 | 0,00777 | 16,01199 |
| IL-6 | 0,16484 | 2,17976 | 0,45004 | 1,65836 | 0,00198 | 253,06825 |
| IL15 | 0,15454 | 1,73922 | 0,00957 | 6,19899 | 0,01712 | 5,60925 |
| IL1RN | 0,16251 | 1,79103 | 0,0783 | 2,43873 | 0,03638 | 3,0107 |
| LEF1 | 0,17556 | -4,75174 | 0,00728 | -52,40798 | 0,62733 | 1,08649 |
| PLAU | 0,03595 | -2,69319 | 0,01119 | 12,97055 | 0,01156 | 16,08562 |
| PSMB9 | 0,49832 | 1,03062 | 0,00734 | 12,72376 | 0,01938 | 8,72541 |
| PTX3 | 0,50824 | -1,37054 | 0,00922 | 21,11118 | 0,01326 | 14,44827 |
| RELB | 0,23565 | 1,48683 | 0,22894 | -1,30584 | 0,05223 | -2,67749 |
| TAP1 | 0,26141 | 1,24808 | 0,00433 | 18,71424 | 0,00403 | 19,27558 |
| TRAF1 | 0,32438 | 2,19423 | 0,01323 | 6,37216 | 0,02126 | 4,78853 |
| VCAM-1 | 0,32253 | 1,07371 | 0,02283 | 445,48301 | 0,0393 | 153,39153 |
| B2M | 0,26953 | 1,12847 | 0,73357 | 4,76118 | 0,44876 | -1,04896 |
| BAX | 0,52788 | -1,15095 | 0,21202 | 1,56711 | 0,19808 | 1,60449 |
| BCL2 | 0,54458 | -1,12849 | 0,45718 | -1,07284 | 0,45138 | -1,16241 |
| BCL2L1 | 0,39804 | -1,32508 | 0,29325 | -1,74045 | 0,38011 | -1,63036 |
| BMP2 | 0,55385 | -1,31391 | 0,42236 | 1,03502 | 0,32687 | -1,45118 |
| BMP4 | 0,55142 | 1,03166 | 0,50789 | -1,11653 | 0,49239 | 1,04702 |
| C4A | 0,32993 | 1,00229 | 0,31539 | -1,61973 | 0,27417 | -1,91515 |
| C4BPA | 0,4232 | -1,46095 | 0,26968 | -1,179 | 0,36188 | 1,05446 |
| CASP4 | 0,20712 | -1,50913 | 0,59421 | 1,62774 | 0,52924 | -1,0259 |
| CCL13 | NS | -1,09989 | 0,18855 | 1,59181 | 0,31898 | -1,23057 |
| CCL4 | 0,32809 | -2,81401 | 0,31785 | 2,55366 | 0,4436 | 1,61117 |
| CCR5 | 0,30178 | -1,41056 | 0,57293 | -1,16531 | 0,58007 | 1,1105 |
| CCR7 | 0,15032 | 1,6847 | 0,53376 | 1,10786 | 0,38913 | 199,50032 |
| CD14 | 0,20308 | 1,69917 | 0,34782 | -1,18246 | 0,42643 | 1,22982 |
| CD209 | 0,25204 | 1,22158 | 0,22822 | 1,34645 | 0,20862 | 1,72839 |
| CD274 | 0,30057 | -1,33368 | 0,23583 | -1,16411 | 0,36445 | 1,26172 |
| CD3G | 0,27831 | -1,52012 | 0,40039 | -1,04407 | 0,37456 | -1,29441 |
| CD40 | 0,35072 | -1,40605 | 0,08255 | 3,63441 | 0,42775 | -1,28182 |
| CD69 | 0,41182 | 1,06541 | 0,32805 | 1,25322 | 0,31777 | -1,348 |
| CD80 | 0,7018 | 1,01709 | 0,29639 | 1,02594 | 0,38546 | 1,31074 |
| CD86 | 0,37169 | -1,31591 | 0,41859 | -1681372916 | 0,46184 | -560546838,3 |
| CFLAR | 0,2547 | 1,21766 | 0,17817 | -1,63929 | 0,45308 | -1,09021 |
| CR2 | NS | 1,07613 | 0,22466 | 1,56123 | 0,24265 | 1,48467 |
| CRP | 0,56012 | -1,36368 | NS | 1,02644 | NS | -1,28431 |
| CTSB | 0,34596 | 1,00304 | 0,60284 | 1,0682 | 0,59687 | -1,05324 |
| CXCL10 | 0,44135 | -1,16341 | 0,23337 | -1,23943 | 0,3567 | -1,1192 |
| CXCL9 | 0,39982 | -1,94197 | 0,07092 | 2,72289 | 0,39694 | 1,04281 |
| DEFB4 | 0,5204 | 1,35752 | 0,34775 | -1,81671 | 0,39156 | -1,8904 |
| F11R | 0,56646 | -1,12689 | 0,5511 | 1,09104 | 0,59096 | 1,52336 |
| F8 | 0,39014 | -1,09623 | 0,26387 | -1,73857 | 0,52675 | -1,11443 |
| FASLG | 0,16933 | -5,78322 | 0,14267 | -1,5895 | 0,28593 | -1,40628 |
| FCER2 | 0,66827 | -1,59525 | 0,72854 | -1,12929 | 0,66289 | -1,23899 |
| GATA3 | 0,37923 | 1,11929 | NS | 1,0223 | 0,30016 | 2,96564 |
| GZMB | 0,37781 | -1,56498 | 0,30418 | -1,47864 | 0,38539 | -1,22474 |
| HLA-DQB2 | 0,41399 | -1,31243 | 0,4902 | -1,17996 | 0,52918 | -1,10173 |
| HLA-DRB1 | 0,45066 | 1,05756 | 0,35715 | 1,35416 | 0,50644 | -1,02509 |
| Hs18s | 0,3741 | -1,0446 | 0,45154 | -1,07416 | 0,38766 | 1,41897 |
| HSGenomic | NS | -1,47423 | 0,3032 | -1,15088 | 0,43982 | 1,08695 |
| ICOS | 0,31035 | -4465,95267 | NS | 1,60656 | NS | -1,21019 |
| IFNB1 | NS | 1,07613 | 0,43074 | -1721,82352 | 0,39898 | -2104,49496 |
| IFNG | 0,36324 | -1,05234 | NS | 12,91187 | NS | 23,49513 |
| IGHE | 0,21266 | 1,54794 | 0,43992 | -1,01084 | 0,50425 | 2,77933 |
| IGHG1 | 0,32633 | 1,21797 | 0,40155 | -1,18456 | 0,40586 | 1,16319 |
| IGK@ | 0,2294 | -1,51118 | 0,23246 | -1,64095 | 0,47745 | -1,02686 |
| IL10 | 0,47823 | 2,04022 | 0,40288 | -1,08238 | 0,40064 | -1,16456 |
| IL12B | 0,09405 | -2,94795 | 0,05529 | -8,69932 | 0,46348 | -1,03015 |
| IL17A | 0,48062 | -1,3584 | 0,22044 | -2,16445 | 0,35653 | -1,39595 |
| IL1B | 0,36606 | -1,19171 | 0,18781 | -2,43916 | 0,4447 | 1,64624 |
| IL2 | 0,40517 | -1,11311 | 0,30677 | 1,33171 | 0,39992 | -1,04024 |
| IL23A | 0,40417 | -1,06158 | 0,40986 | -1,05865 | 0,20634 | -1,61464 |
| IL27 | 0,5496 | -1,01382 | 0,30344 | 1,48709 | 0,38371 | 1,35314 |
| IL2RA | 0,37623 | 25,11254 | 0,4314 | 1,93652 | 0,56066 | 1,25758 |
| IRF1 | 0,47098 | -1,04865 | NS | -4,40245 | 0,28546 | 5,80652 |
| IRF4 | NS | -1,24302 | 0,55928 | 1,12717 | 0,61838 | -1,03692 |
| KIT | 0,34655 | -1,45282 | 0,45298 | 1,02266 | 0,23603 | -1,59238 |
| LTA | 0,36494 | -1,16408 | 0,21207 | 1,65589 | 0,40072 | 1,03625 |
| MMP9 | 0,35462 | -1,19169 | 0,11831 | 3,22212 | 0,38686 | 1,3228 |
| NOD2 | 0,15296 | 1,80679 | 0,27621 | 1,53209 | 0,26749 | 1,60498 |
| NOS2 | 0,49268 | -1,11186 | 0,64298 | 4,27701 | 0,50276 | 2,11085 |
| PRF1 | 0,1215 | -2,54544 | 0,1234 | -2,17436 | 0,3969 | -1,25636 |
| PRKCD | 0,32522 | -1,33457 | 0,35547 | -1,01598 | 0,29624 | -1,36265 |
| RAG1 | 0,27355 | -1,63199 | 0,46146 | 1,2891 | 0,57276 | -1,0237 |
| SELP | NS | -1,61546 | NS | -2,20566 | NS | -1,33861 |
| SERPINE1 | 0,35935 | -1,5223 | 0,45274 | -1,18474 | 0,26862 | -1,84532 |
| SOD1 | 0,29649 | 1,40057 | 0,49633 | 1,04682 | 0,38629 | 1,30595 |
| STAT5A | 0,49275 | -1,47226 | 0,41574 | -1,45126 | 0,53229 | -1,16053 |
| TICAM1 | 0,45269 | -1,03311 | 0,34643 | 1,4637 | 0,28193 | 1,47089 |
| TLR2 | 0,57944 | -1,18028 | 0,47159 | 1,57141 | 0,17877 | -2,3562 |
| TLR9 | 0,50934 | -1,05683 | 0,50816 | -1,11272 | 0,25859 | 2,23977 |
| TNF | 0,36311 | 1,01942 | 0,07813 | 2,97554 | 0,28367 | 1,84911 |
| TNFRSF1B | 0,31783 | 1,4954 | 0,20937 | 1,51377 | 0,07975 | 3,48164 |
| TNFSF10 | 0,43688 | -1,09669 | 0,25131 | 1,74311 | 0,51092 | 1,04728 |
| TNFSF13B | 0,23264 | -1,81134 | 0,28442 | -1,18384 | 0,05541 | -2,467 |
| TRAF2 | 0,52964 | 1,01525 | 0,30146 | 1,67853 | 0,35562 | 1,40836 |

Table2: primer sequences used for RT-qPCR.

| **gene** | **frw** | **rev** |
| --- | --- | --- |
| hIL-6 | GACAGCCACTCACCTCTTCA | AGTGCCTCTTTGCTGCTTTC |
| hCXCL-2 | CCCATGGTTAAGAAAATCATCG | CTTCAGGAACAGCCACCAAT |
| hCCL-5 | TGCCCACATCAAGGAGTATTT | TTTCGGGTGACAAAGACGA |
| hICAM-1 | GCAGACAGTGACCATCTACAGCTT | CTTCTGAGACCTCTGGCTTCGT |
| hVCAM-1 | TCTCATTGACTTGCAGCACC | TTCTTGCAGCTTTGTGGATG |
| hA20 | CCTTGCTTTGAGTCAGGCTGT | AAGGAGAAGCACGAAACATC |
| hC3 | CTGCTGCTCCTGCTACTAAC | TCGTGGACAGTAACAGTGAC |
| hCXCL-3 | AAAATCATCGAAAAGATACTGAACAA | GGTAAGGGCAGGGACCAC |
| hHPRT | TGACACTGGCAAAACAATGCA | GGTCCTTTTCACCAGCAAGCT |
| rIL-6 | GGAGTGCTAAGGACCAAGACCA | AGGTTTGCCGAGTAGACCTCA |
| rCXCL-2 | TTCTCGGGGCTTACAGAAAA | AGGGGGAGTTGGGTACTGAC |
| rCCL-5 | GAGTAGGGGGTTGCTCAGTG | GCCAACCCAGAGAAGAAGTG |
| rICAM-1 | CTCCGTGGGAATGAGACACT | TTGAACAGTGACAGCCCTTG |
| rVCAM-1 | CAAATGGAGTCTGAACCCAAA | GGTTCTTTCGGAGCAACG |
| rA20 | TGCATGCAGCCTGTCAGTA | CCTTCCTCAGGACCAAGTCA |
| rC3 | TCGAAATCCCTCCCAAGTC | CGATCTTCAAGGGGACAATG |
| rCXCL-3 | TCACTTCCATTCTGTTGCAG | CCTCCCTGTGACACTGAAGA |
| rHPRT | CTCATGGACTGATTATGGACAGGAC | GCAGGTCAGCAAAGAACTTATAGCC |

Fig 1: representative plots for lymphocyte gating.


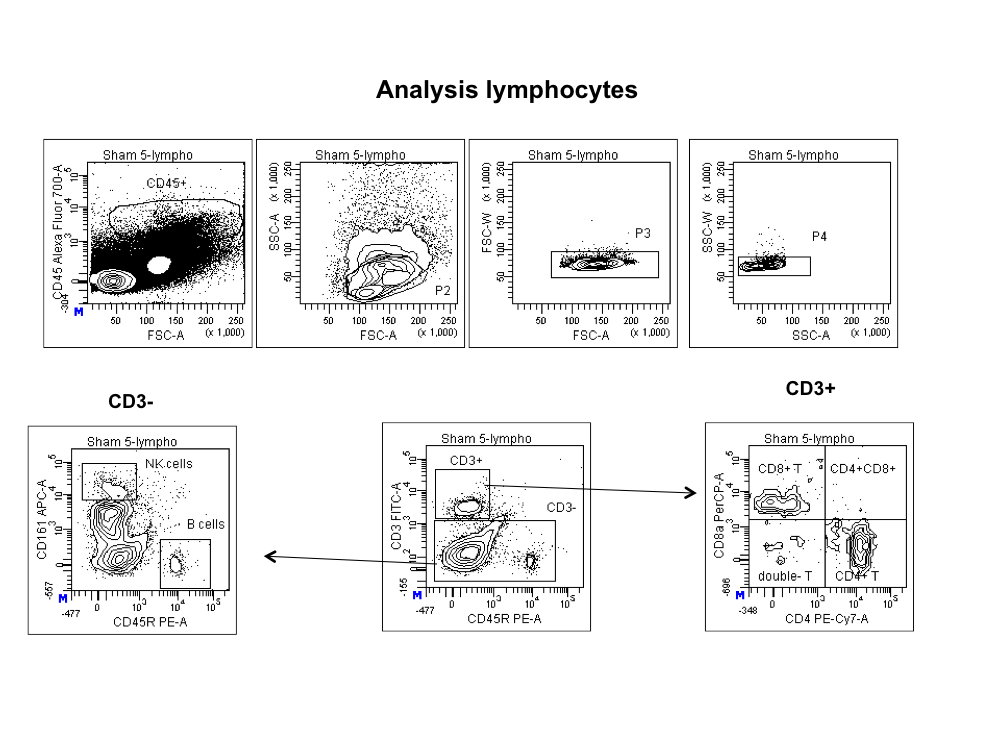


Fig 2: representative plots for myeloid gating.


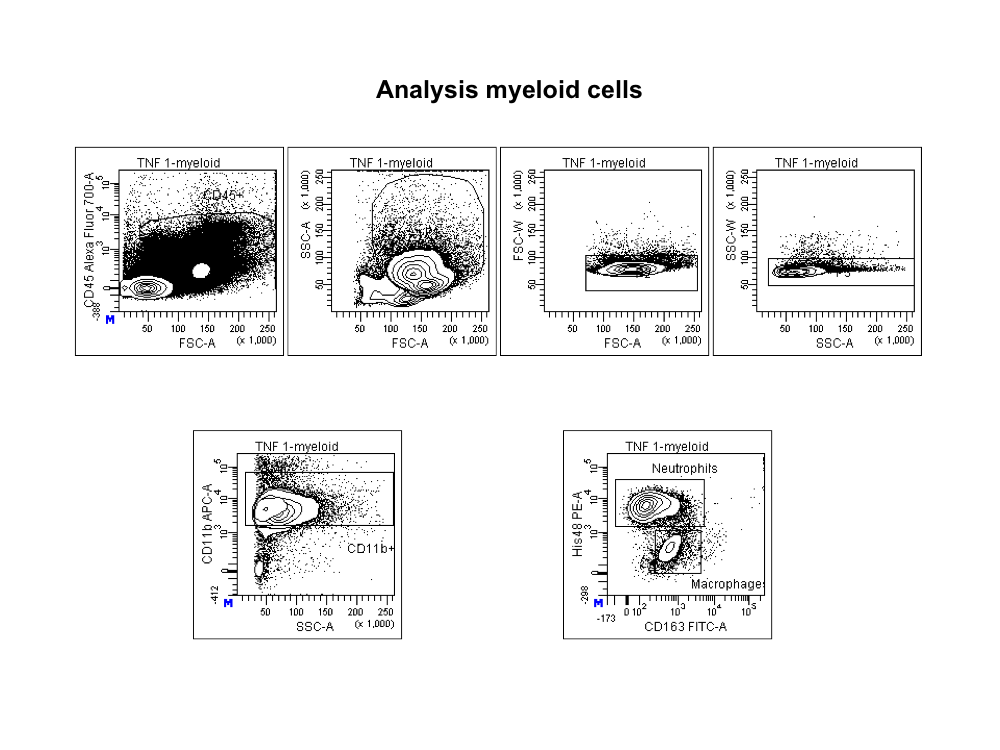


Figure 3: Leucocyte subsets plotted as median with interquartile range for the different treatment conditions. Statistical analysis was performed with a Kruskal-Wallis test with Dunn’s post-hoc for multiple comparisons (* = P<0.05). Abbreviations: Natural Killer (NK) cells, Natural Killer T (NKT) cells, CD4+CD8+ double positive (DP) T cells, CD4-CD8- double negative (DN) T cells.


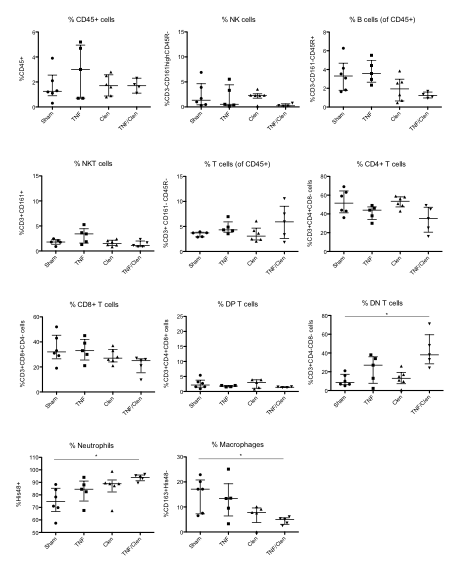

Supplement: Additional file 1 — Table S1. Genes screened for in the qPCR-array for each treatment condition. Significant changes in upregulation (P <0.05) are marked in red, significant downregulation in green (P <0.05), and unchanged genes in yellow versus vehicle. For clarity, genes with a significant change have been put alphabetically at the beginning of the table followed by unaltered genes in alphabetical order. Table S2. Primer sequences used for RT-qPCR. Figure S1. Representative plots illustrating lymphocyte gating. Figure S2. Representative plots illustrating myeloid gating. Figure S3. Leucocyte subsets plotted as median with interquartile range for the different treatment conditions. Statistical analysis was performed with a Kruskal-Wallis test with Dunn’s post-hoc for multiple comparisons (* = P < 0.05). Abbreviations: Natural Killer (NK) cells, Natural Killer T (NKT) cells, CD4 + CD8+ double positive (DP) T cells, CD4-CD8- double negative (DN) T cells. [file 1742-2094-11-21-S1.docx]
